# Liver-eye axis in metabolic syndrome: integrated evidence from mendelian randomization, single-cell sequencing, and clinical sample validation

**DOI:** 10.3389/fendo.2026.1878316

**Published:** 2026-09-09

**Authors:** Tian-Hao Yuan, Yi-Fan Fang, Guo-Heng Zhang, Hao-Rui Zhang, Zi-Yi Zhou, Zhen-Sheng Yue, Bei Li, Lin Wang, Yu-Sheng Wang, Guo-Rui Dou

**Affiliations:** 1 Department of Ophthalmology, Eye Institute of Chinese People’s Liberation Army (PLA), Xijing Hospital, Fourth Military Medical University, Xi’an, Shaanxi, China; 2 Department of Hepatobiliary Surgery, Xi-Jing Hospital, Fourth Military Medical University, Xi’an, Shaanxi, China; 3 State Key Laboratory of Oral and Maxillofacial Reconstruction and Regeneration, National Clinical Research Center for Oral Disease, Shaanxi International Joint Research Center for Oral Diseases, Center for Tissue Engineering, School of Stomatology, Fourth Military Medical University, Xi’an, Shaanxi, China

**Keywords:** blood proteomics, diabetic retinopathy, liver-eye axis, lymphotoxin β, non-alcoholic fatty liver disease, organ interaction

## Abstract

**Background:**

Non-alcoholic fatty liver disease (NAFLD) and diabetic retinopathy (DR) are common clinical complications of metabolic syndrome in different organ systems. However, the relationship between NAFLD and DR remains controversial, and more high-level evidence is needed to clarify the causal link between them. We aimed to investigate the causal relationship between NAFLD and DR across its progression stages, and to identify circulating proteins may mediate this association.

**Materials and methods:**

Based on genetic tools, a multi-stage network MR framework was constructed, integrating NAFLD/DR genome-wide association study data and three blood proteome-wide datasets. Causal effects of NAFLD on DR were assessed using inverse-variance weighted regression and multimodal models. Proteome-wide MR identified DR-associated proteins. Bidirectional two-sample MR and mediation models quantified regulatory weights in NAFLD-protein-DR pathways. In addition, the transcriptional expression levels of the identified mediator proteins in the liver were verified through single-cell transcriptome sequencing. The levels of the mediator protein in the serum samples of clinical patients were detected by ELISA. The levels of mediator proteins in the aqueous humor and vitreous humor samples of clinical patients were detected by Luminex liquid-phase protein chip.

**Results:**

NAFLD increased DR risk (OR = 1.04, 95% CI: 1.01-1.08) and specifically promoted non-proliferative DR (NPDR) progression (OR = 1.26, 95% CI: 1.05-1.52). Four proteins were identified as candidate mediators of NAFLD-DR association, among them, LTB (lymphotoxin-β, a TNF superfamily member involved in immune regulation and inflammatory responses) showed the strongest effect. In the liver of NAFLD model mice, the expression of the LTB gene was mainly elevated in T cells and B cells. The levels of LTB protein in the aqueous humor, vitreous humor and serum of NAFLD patients with DR were all higher than those of NAFLD patients without DR.

**Conclusions:**

NAFLD is causally associated with an increased risk of DR development, particularly during early NPDR stages. LTB may mediate the biological process by which NAFLD promotes DR. T cells and B cells in the liver of patients with NAFLD produce higher levels of LTB protein. In NAFLD patients with DR, LTB protein levels were elevated in serum, aqueous humor, and vitreous humor compared to NAFLD patients without DR, suggesting that LTB may play a role in the link between these two conditions.

## 1 Introduction

Non-alcoholic fatty liver disease (NAFLD), also referred to as metabolic dysfunction-associated fatty liver disease (MAFLD), represents the most prevalent hepatic complication associated with metabolic syndrome. Over the past two decades, NAFLD has rapidly emerged as the most common chronic liver disease globally, affecting approximately 25% of the world’s population (1). It should be noted that, as one of the most prominent disorders, NAFLD is not merely a condition coexisting with metabolic vascular complications but also an independent risk factor and a critical driver for their development and progression, according to substantial body of epidemiological, clinical, and basic research (2). Among these metabolic complications, diabetic retinopathy (DR), a severe blinding ocular complication of metabolic syndrome, constitutes a leading cause of vision loss in developed countries (3). Indeed, the relationship of NAFLD-DR has been reported by a few studies including cross-sectional study and case-control study (4, 5), but the definite conclusion is still absent. Our previous meta-analysis demonstrated that liver fibrosis, rather than NAFLD per se, was significantly associated with DR, particularly in type 1 diabetes patients, suggesting a complex relationship between hepatic and retinal pathologies that warrants further mechanistic exploration (6). Thus more sufficient evidence needed to be exhibited and the underling mechanism should be unveiled.

The liver, serving as a central hub in metabolism, participates in diverse functions including endocrine regulation, immune modulation, and storage, thereby communicating with distant organs through these mechanisms to regulate pathophysiological processes. Emerging evidence indicates that liver-centered interorgan communication play essential roles in several diseases such as hepatic osteodystrophy (7), myocardial infarction (8) and anxiety (9), which are possibly mediated through communicating molecules derived from the liver (10). Thus, a liver-eye axis may mediate the pathophysiological processes between NAFLD and DR, a possibility that warrants further investigation. The endeavor to study liver-eye axis will contribute to providing novel perspectives on multi-organ complications such as NAFLD and DR in metabolic diseases, as well as offering new risk stratification and biomarkers for the diagnosis and treatment of NAFLD and DR.

Emerging Mendelian randomization (MR) analysis methodologies based on genetic tools offer a promising solution to this demand. MR leverages the random assortment of genetic variants at conception—analogous to the randomization in a controlled trial—to estimate causal effects between exposures and outcomes. By utilizing highly stable single nucleotide polymorphisms (SNPs) as genetic markers, MR effectively addresses the inherent residual confounding and bias issues in observational studies. However, valid MR inference relies on three core assumptions: (1) the IVs are robustly associated with the exposure; (2) the IVs are independent of confounders; and (3) the IVs affect the outcome only through the exposure. The robustness of MR findings can be further assessed through sensitivity analyses, including tests for horizontal pleiotropy (e.g., MR-Egger intercept and MR-PRESSO global test), heterogeneity assessment (Cochran’s Q), and the Steiger directionality test for causal direction, which collectively help evaluate the validity of the causal estimates. For our research question, MR is particularly well-suited because it enables causal inference between NAFLD and DR without requiring both conditions to be assessed within the same cohort—an advantage given that NAFLD and DR are often not concomitantly evaluated in large-scale epidemiological studies. Additionally, integrating MR with protein quantitative trait loci (pQTL) data allows us to systematically screen for circulating proteins that may mediate the NAFLD-DR association, offering a cost-effective strategy to prioritize candidate mediators for further experimental investigation.

In summary, elucidating the existence and interaction mechanisms of the liver-eye axis represented by NAFLD and DR in metabolic syndrome not only facilitates the interpretation of multi-organ pathophysiological interactions underlying metabolic dysregulation but also advances the holistic understanding of metabolic diseases. Consequently, we conducted this study to investigate the causal relationship between NAFLD and DR through MR based on genetic tools, and to elucidate the protein networks that may link these two diseases.

## 2 Materials and methods

### 2.1 Study design

In this study, we included NAFLD and DR, and diabetic retinopathy was further stratified into non-proliferative (NPDR) and proliferative (PDR) stages for subgroup analysis. The network MR analysis involved three steps. First, we examined the association between NAFLD and DR, with additional subgroup analyses separating DR into NPDR and PDR stages. In the second step, we performed proteome-wide MR analyses across three blood protein genome-wide association study (GWAS) datasets to investigate associations between genetic predisposition to DR and blood protein levels. After that, we evaluated whether all significantly DR-associated blood proteins identified in the second step could be causally influenced by NAFLD. Additionally, we conducted several supplementary analyses, including genetic colocalization analysis and mediation effect analysis, to further clarify the potential role of identified blood proteins in mediating the interaction between the two diseases.

### 2.2 Study populations and data sources

#### 2.2.1 NAFLD-related disease data and genetic variants

NAFLD-related disease data and genetic instruments were obtained from the FinnGen R12 database, a GWAS that sampled nearly 10% of the Finnish population, comprising over 500,000 genotyped and phenotyped individuals (11), with high evidence-based medicine credibility. The NAFLD-related cohort included 496,844 control individuals and 8,434 case individuals, with more detailed information provided in **Supplementary Table 1**. We used strict selection criteria to obtain valid instrumental variables (IVs): NAFLD-associated genetic variants (e.g. SNPs) met genome-wide significance (p < 5×10^-8^) and were pruned for linkage disequilibrium (r^2^ < 0.001 within a 10–000 kb window) to ensure independence. SNPs associated with potential confounders were excluded to comply with the second MR assumption of IV-confounder independence, and to avoid the occurrence of weak instrumental variable bias, instrumental variables with an F-statistic greater than 10 are selected (12–14). Ultimately, 27, 24 and 23 independent SNPs were retained, respectively, for the subsequent MR analyses of NAFLD with DR, NPDR and PDR.

#### 2.2.2 Blood proteomic data

To identify the association between DR susceptibility and blood protein levels, blood protein level data were obtained from three proteome-wide association study datasets with no sample overlap. Among these, the Decode proteomic genetic database examined associations between 27 million sequence variants and 4,719 protein levels in blood samples from 35,559 Icelandic individuals (15). Additionally, data on 2,923 blood protein levels from 54,219 independent individuals in the UK Biobank Pharma Proteomics Project (UKB-PPP) were included in our study (16). The Fenland Study is a genome-proteome-wide association study of 4,775 distinct proteins measured in plasma samples from 10,708 generally healthy individuals European ancestry (17). More detailed information on the protein GWAS databases used is provided in **Supplementary Table 1**. The Decode database and Fenland Study utilized the SomaScan version 4 assay platform, while UKB-PPP employed the Olink Explore 3072 PEA platform. Furthermore, for each protein, we selected SNPs located within ±1 Mb of its corresponding gene transcription start site (i.e., cis-pQTL) as the genetic instrument. To ensure independence of the genetic instruments, we performed linkage disequilibrium (r^2^ < 0.001 within a 10,000 kb window). Instrument strength was evaluated using the F-statistic, and SNPs with F < 10 were excluded to avoid weak instrument bias. These selected SNPs were then used as genetic instruments for the subsequent proteomic MR analyses.

#### 2.2.3 DR-related disease data and genetic variants

The DR-related disease data used in our study were sourced from the same FinnGen database as the NAFLD data to ensure consistency in subsequent processing of disease data and genetic variables between the two distinct conditions (11). We emphasize that in the Mendelian randomization framework, the DR GWAS data were used to extract SNP-outcome associations, rather than serving as exposure instruments. The overall DR data were obtained from the final FinnGen R12 release, comprising 82,287 control individuals and 14,142 case individuals. Additionally, since DR can be clinically categorized into non-proliferative and proliferative stages (NPDR and PDR), we utilized the “Severe non-proliferative background diabetic retinopathy” dataset from FinnGen R7 and the “Proliferative diabetic retinopathy” dataset from FinnGen R9 to acquire more detailed research data. Of note, the NPDR and PDR classifications were pre-defined within the respective FinnGen datasets. Comprehensive information on these datasets is provided in **Supplementary Table 1**.

### 2.3 Statistical analysis

#### 2.3.1 Two-sample mendelian randomization analysis

In the two-sample Mendelian randomization (TSMR) analysis for both diseases, we treated NAFLD as the exposure and proliferative diabetic retinopathy/severe non-proliferative background diabetic retinopathy as outcomes to investigate whether NAFLD acts as a risk factor for these conditions. The inverse-variance weighted (IVW) linear regression method was employed to estimate the causal effects of NAFLD on DR and its distinct stages. The IVW method provides unbiased causal estimates under the assumption that all genetic instruments are valid and there is no directional horizontal pleiotropy (or that pleiotropy is balanced under certain conditions) (18). To assess potential horizontal pleiotropy and heterogeneity, we calculated intercepts and P-values through MR-Egger regression analysis as sensitivity indicators for horizontal pleiotropy. And when the p-value of the intercept of the MR-Egger intercept is greater than 0.05, indicated no evidence of directional horizontal pleiotropy (19). Additionally, Cochran’s Q statistic was used to evaluate heterogeneity among IVs, with P < 0.05 indicating significant heterogeneity (20). Four complementary MR methods MR-Egger, Simple mode, Weighted median, and Weighted mode (21–23) were implemented as sensitivity analyses to ensure result robustness and address potential pleiotropic effects of selected IVs. Additionally, the MR-PRESSO (Mendelian Randomization Pleiotropy RESidual Sum and Outlier) global test was performed to detect horizontal pleiotropy and identify potential outlier SNPs, with a P-value < 0.05 indicating significant overall pleiotropy. The Steiger directionality test was also conducted to verify the presumed causal direction from NAFLD to DR, by comparing the variance explained by the genetic instruments in the exposure versus the outcome. Multiple testing correction was performed using the Benjamini-Hochberg false discovery rate (FDR) method (24), with adjusted FDR < 0.05 considered statistically significant for causal associations. All tests were two-sided. All analyses were performed in R 4.3.2 (R Foundation for Statistical Computing) using the TwoSampleMR package (v0.5.9).

#### 2.3.2 Proteome-wide mendelian randomization analysis

In the proteome-wide Mendelian randomization analysis, we used three pQTL datasets as exposure data and severe non-proliferative background diabetic retinopathy/diabetic retinopathy as outcome data to perform TSMR, identifying blood proteins causally linked to DR. Subsequently, NAFLD was treated as the exposure data and the DR associated effector proteins identified in the first step as the outcome data TSMR analysis, further validating mediator proteins whose expression levels could be driven by NAFLD. The Wald ratio method served as the primary analytical approach, as it is the standard method for estimating causal effects when a single genetic instrument is available for a given exposure (25). In our proteomic MR analysis, many proteins were instrumented by only one cis-pQTL after LD pruning, making the Wald ratio the most appropriate and consistent estimator across all protein analyses. For proteins with multiple independent cis-pQTLs, we additionally applied IVW, MR-Egger, Simple mode, Weighted median, and Weighted mode methods as complementary sensitivity analyses to ensure robustness. Consistent with the two-sample MR analysis protocol, multiple testing correction was implemented using the Benjamini-Hochberg FDR method (24), with adjusted FDR < 0.05 considered statistically significant for causal relationships. All tests were two-sided. All analyses were performed in R 4.3.2 (R Foundation for Statistical Computing) using the TwoSampleMR package (v0.5.9).

#### 2.3.3 Colocalization analysis

To investigate whether proteins and DR colocalize at the same genomic loci and strengthen evidence for their associations, we performed genetic Bayesian colocalization analysis. For each protein showing evidence of association with DR, we selected SNPs within 500 kb regions surrounding their cis-pQTLs. Five mutually exclusive hypotheses were defined as follows: (1) Neither blood protein nor DR shows evidence of association signals with any SNPs in a genomic region (PH0); (2) Blood protein shows no evidence of association signals with any SNPs in a genomic region (PH1); (3) DR shows no evidence of association signals with any SNPs in a genomic region (PH2); (4) Both blood protein and DR show evidence of association signals with SNPs in a genomic region but are driven by distinct causal variants (PH3); (5) Both blood protein and DR show evidence of association signals with SNPs in a genomic region and the signals colocalize at the same putative causal variant (PH4). We defined PH4≥0.8 as strong evidence for colocalization, 0.5≤ PH4<0.8 as moderate evidence, and PH4<0.5 as weak evidence.

#### 2.3.4 Mediation mendelian randomization

In this study, to evaluate the mediation proportion of each blood protein in the NAFLD-DR association, we employed mediation Mendelian randomization analysis, treating proteins as potential intermediate factors linking the two phenotypes. The indirect effect was estimated using the coefficient product method (β_NAFLD-protein_ × β_protein-DR_), and the mediation proportion was calculated as the indirect effect divided by the total effect (Mediation proportion = (β_NAFLD-protein_ × β_protein-DR_)/β_NAFLD-DR_). Only proteins with significant associations in both the NAFLD-protein and protein-DR MR analyses (FDR < 0.05) were included in the mediation analysis, and the directionality of the effects was required to be consistent across the total and indirect pathways.

### 2.4 Acquisition and preprocessing of single-cell transcriptome data

Single-cell transcriptomic data were obtained from the GEO database (accession number GSE129516), including three NASH mouse samples and three normal mouse samples. We used the “Read10X” function from the R package “Seurat” to load the single-cell data, followed by the “CreateSeuratObject” function to create a Seurat object. The following filtering criteria were applied: (1) genes expressed in fewer than three cells were excluded; (2) cells with feature counts (nFeature_RNA) between 200 and 5,000 and UMI counts (nCount_RNA) between 200 and 30,000 were retained; and (3) cells with fewer than 200 detected genes were removed. The “IntegrateLayers” function was then applied using the CCA method to correct for batch effects arising during sequencing. Subsequent analyses included the calculation of the percentage of mitochondrial and red blood cell gene expression per cell using the “PercentageFeatureSet” function. Red blood cell genes were identified using the pattern “^HBA|^HBB”, and mitochondrial genes using the pattern “^MT-”. Cells with mitochondrial gene expression exceeding 30% or red blood cell gene expression exceeding 5% were excluded. Data normalization was performed using the “NormalizeData” function, which involved dividing the counts for each gene by the total counts per cell, multiplying by 10,000, and applying a log transformation. Variable features were identified using the “FindVariableFeatures” function, which selects genes exhibiting high variability across cells. By default, the top 2000 highly variable genes were retained. Data scaling was conducted using the “ScaleData” function, followed by dimensionality reduction via principal component analysis (PCA) using the “RunPCA” function with the first 15 principal components retained for downstream analysis. After PCA, unsupervised clustering was performed using the “FindNeighbors” and “FindClusters” functions from the Seurat package. Based on the cell count in this study and relevant literature, a resolution of 1 was chosen, yielding 17 clusters for downstream analysis. Uniform manifold approximation and projection (UMAP) was applied using the “RunUMAP” function for visualization. Cell annotation was performed using the SingleR package with a mouse RNA-seq reference dataset. Based on the annotated cell types, this study focused on the expression of the LTB gene across different cell types. Statistical significance of gene expression was assessed using the Wilcoxon rank-sum test.

### 2.5 Clinical sample collection

Plasma, aqueous humor, and vitreous humor samples were collected from patients diagnosed with NAFLD with or without DR at Xijing Hospital. The diagnosis of NAFLD was established based on standard clinical criteria: ultrasonographic detection of hepatic steatosis after exclusion of other liver diseases, with hepatic steatosis graded as mild (5%–10% hepatic fat content), moderate (10%–25%), or severe (>25%) according to ultrasound findings. DR was diagnosed and staged by experienced ophthalmologists according to standard clinical protocols, using the International Clinical Diabetic Retinopathy (ICDR) Disease Severity Scale based on fundus photography and optical coherence tomography. DR severity was classified into three categories: no DR (no retinal abnormalities), non-proliferative DR (NPDR; characterized by microaneurysms, hemorrhages, exudates, and/or intraretinal microvascular abnormalities without neovascularization), and proliferative DR (PDR; characterized by neovascularization and/or vitreous/preretinal hemorrhage). Exclusion criteria included other liver diseases (viral hepatitis, alcoholic liver disease, autoimmune hepatitis), other ocular diseases (glaucoma, age-related macular degeneration, retinal vein occlusion), and history of ocular surgery or laser treatment. A total of 14 patients were included in this study, consisting of 8 NAFLD patients with PDR who underwent vitrectomy surgery and 6 NAFLD patients without DR who had idiopathic epiretinal membrane and underwent vitrectomy as controls. The detailed information of the research participants is provided in **Supplementary Table 10**. All participants provided written informed consent, and the study protocol was approved by the Institutional Review Board of Xijing Hospital.

### 2.6 Measurement of LTB protein levels by luminex assay

The concentrations of LTB protein in aqueous humor and vitreous fluid samples were quantified using a custom magnetic Luminex xMAP assay. Briefly, all samples were centrifuged at 10,000 × g for 10 minutes at 4 °C to remove any insoluble debris or cells. The clarified supernatants were collected and analyzed undiluted. A pre-mixed magnetic bead set, conjugated with specific capture antibodies against LTB, was used according to the manufacturer’s instructions. Standards, quality controls, and samples were added in duplicate to a 96-well plate containing the magnetic beads and incubated overnight on a plate shaker at 4 °C. Following a series of washes, a biotinylated detection antibody was added to form a sandwich complex. Subsequently, the mixture was incubated with streptavidin-phycoerythrin for signal amplification. The plate was then read on a Luminex MAGPIX^®^ or FLEXMAP 3D^®^ instrument. The concentration of LTB in each sample was determined by interpolating the median fluorescence intensity from the standard curve generated using the provided recombinant proteins. Data analysis was performed using the instrument’s xPONENT^®^ software and Milliplex^®^ Analyst software, with results expressed in picograms per milliliter (pg/mL).

### 2.7 Measurement of serum LTB by enzyme-linked immunosorbent assay

The concentration of LTB in human plasma samples was quantified using a commercial sandwich ELISA kit (Elabscience, Catalog No: ELK2068) in accordance with the manufacturer’s protocol. Briefly, venous blood was collected from participants into anticoagulant tubes. The samples were centrifuged at 3,000 × g for 10 minutes at 4 °C to separate plasma, which was then aliquoted and stored at -80 °C until analysis. All reagents, standards, and plasma samples were equilibrated to room temperature prior to the assay. A 100 μL aliquot of each standard, control, and pre-diluted plasma sample was added to the corresponding wells of the antibody-pre-coated microplate. After sealing, the plate was incubated for 90 minutes at 37 °C. The liquid was then aspirated, and the plate was washed four times with the provided wash buffer. Next, 100 μL of biotinylated detection antibody was added to each well, followed by a 60-minute incubation at 37 °C. After another wash cycle, 100 μL of Horseradish Peroxidase-conjugated streptavidin was added, and the plate was incubated for 30 minutes at 37 °C in the dark. The plate was washed again, and 90 μL of TMB substrate solution was added to each well for a 15-minute incubation at 37 °C in the dark to allow color development. The enzymatic reaction was terminated by adding 50 μL of stop solution. The optical density (OD) of each well was measured immediately at 450 nm using a microplate reader. The concentration of LTB in each sample was calculated by interpolating its average OD value from the standard curve generated with the kit’s recombinant protein standards. All samples were assayed in duplicate, and results are expressed in picograms per milliliter (pg/mL).

## 3 Results

**Figure 1:**
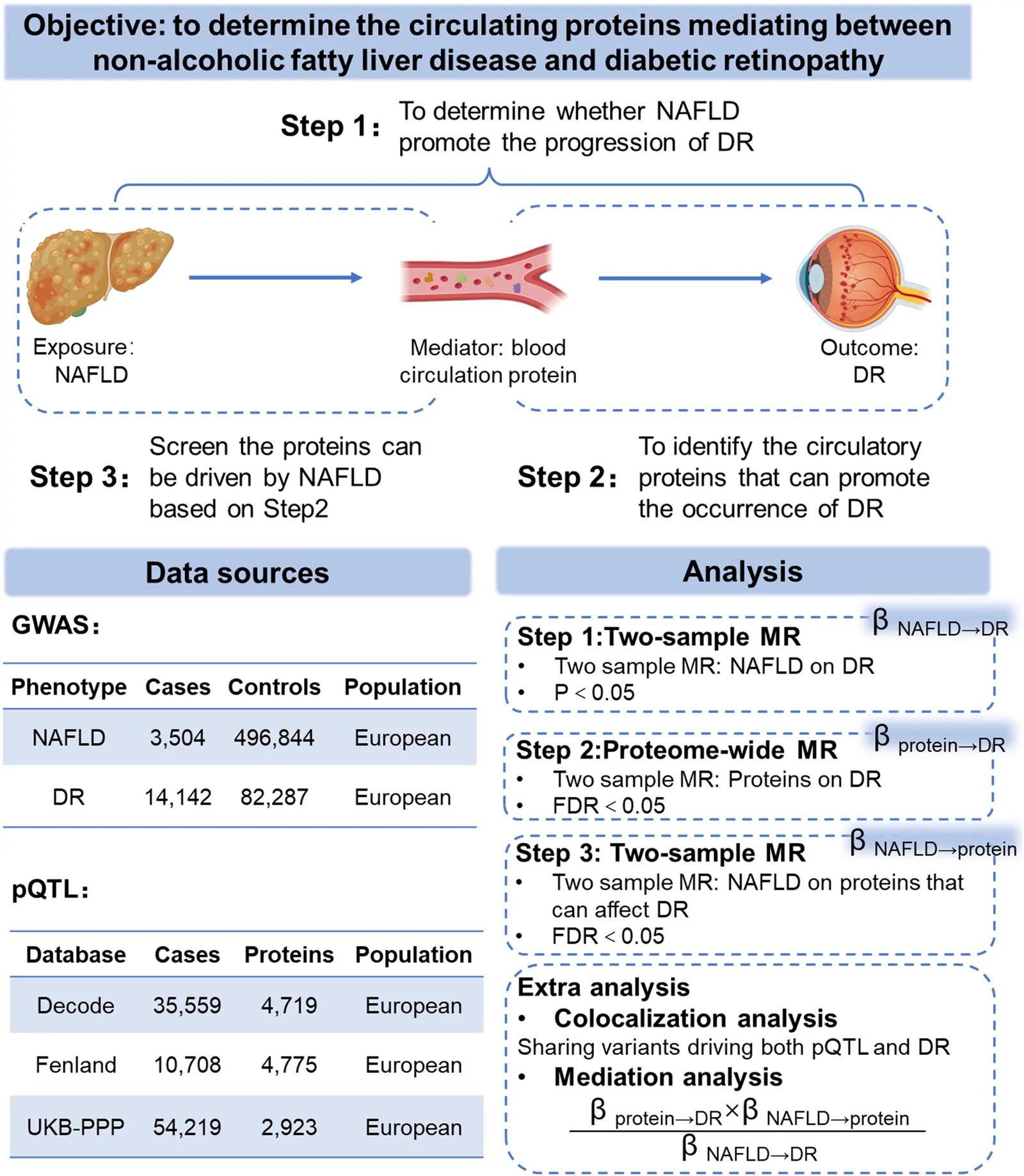
The overall flowchart of the research. NAFLD, non-alcoholic fatty liver disease; DR, diabetic retinopathy;GWAS, genome wide association study; pQTL, protein quantitative trait loci;MR, Mendel randomization.

Our overall research findings are presented in **Figure 1**. Through a three-step two-sample Mendelian randomization, we identified a liver-eye axis signaling pathway mediated by plasma proteins. And this pathway was further validated through biological means.

### 3.1 Genetically predicted NAFLD as a risk factor for DR and NPDR in metabolic syndrome

**Figure 2:**
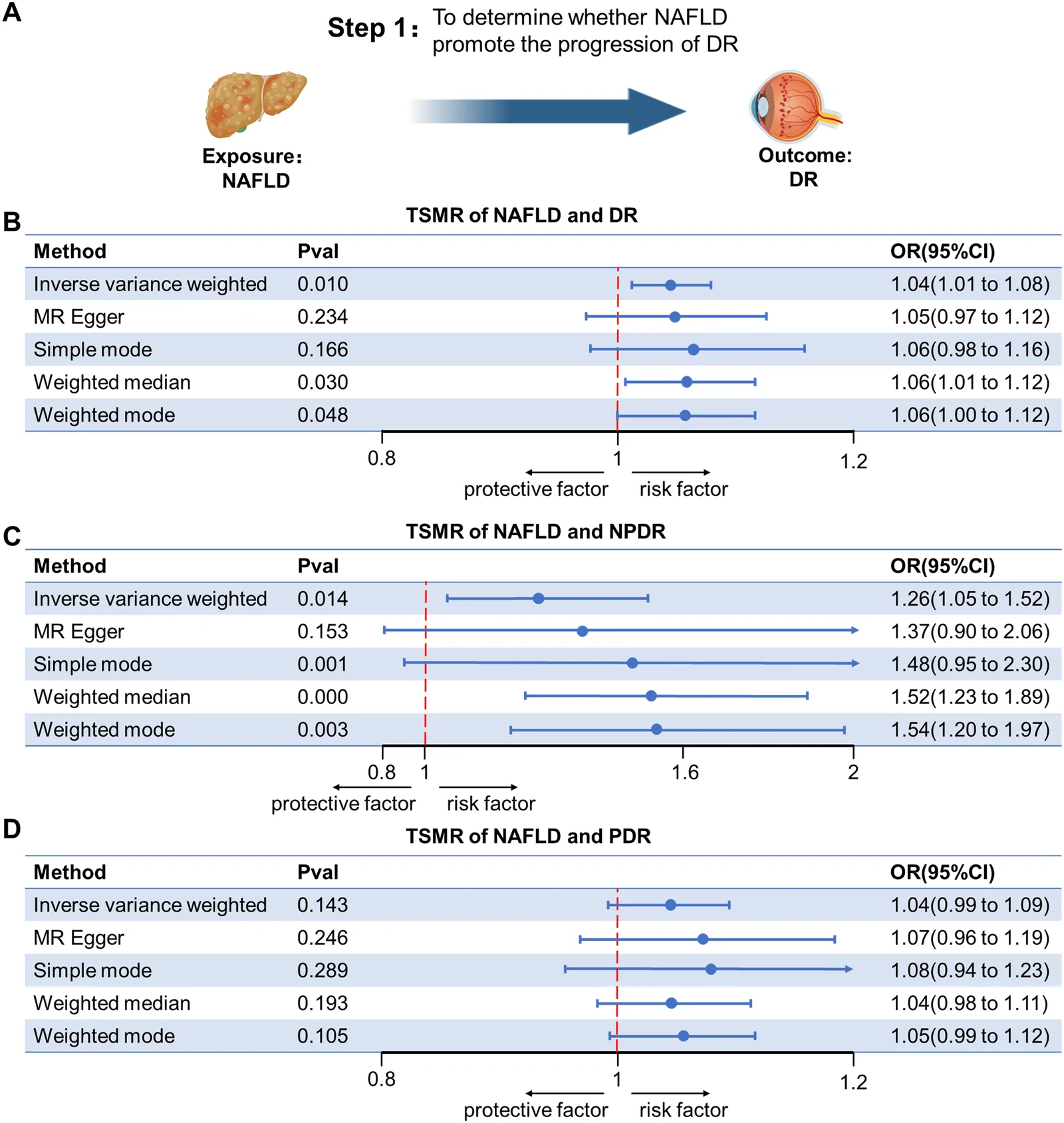
Associations between genetically predicted NAFLD and DR at different stages. **(A)** Schematic diagram of exposure and outcome in two-sample Mendelian randomization. **(B)** Results of TSMR analysis between NAFLD and DR. **(C)** Results of TSMR analysis between NAFLD and NPDR. **(D)** Results of TSMR analysis between NAFLD and PDR. NAFLD, non-alcoholic fatty liver disease; DR, diabetic retinopathy; PDR, proliferative diabetic retinopathy; NPDR, non-proliferative background diabetic retinopathy; CI, confidence interval; OR, odds ratio.

Based on the Mendelian randomization framework, a causal association between NAFLD and DR, which are representative diseases of the liver and eyes in metabolic syndrome, was identified (**Figure 2A**). IVW analysis demonstrated a significant association between genetically predicted NAFLD and DR development, indicating NAFLD as a risk factor for DR (P < 0.05, OR = 1.04, 95% CI: 1.01-1.08). These findings were replicated in Weighted median and Weighted mode analyses, consistently showing that NAFLD promotes DR occurrence (**Figure 2B**). The Cochran’s Q statistic (Q = 23.96, p = 0.58) from IVW indicated low heterogeneity and high reliability. MR-Egger regression revealed no evidence of horizontal pleiotropy (intercept = -0.0002, p = 0.98) (**Supplementary Table 2**).

In subgroup analyses of NPDR and PDR populations, IVW analysis identified NAFLD as a risk factor for NPDR (P < 0.05, OR = 1.26, 95% CI: 1.05-1.52), with consistent results observed in Weighted median and Weighted mode methods (**Figure 2C**). However, no statistically significant association was found between NAFLD and PDR across all five models (P = 0.14, OR = 1.04, 95% CI: 0.99-1.09) (**Figure 2D**). Sensitivity analysis confirmed low heterogeneity (Q = 37.9, p = 0.26) and absence of horizontal pleiotropy for the NPDR findings (**Supplementary Table 2**). The MR-PRESSO global test further confirmed the robustness of the main findings, showing no evidence of significant horizontal pleiotropy (RSSobs = 4.90, P = 0.764) and no outlier SNPs detected for the NAFLD→DR analysis (**Supplementary Table 3**). The Steiger directionality test supported the presumed causal direction from NAFLD to DR, as the genetic instruments explained substantially more variance in the exposure (R^2^ = 0.173) than in the outcome (R^2^ = 0.0013) (**Supplementary Table 3**).

### 3.2 Analysis of blood proteins associated with DR susceptibility

**Figure 3:**
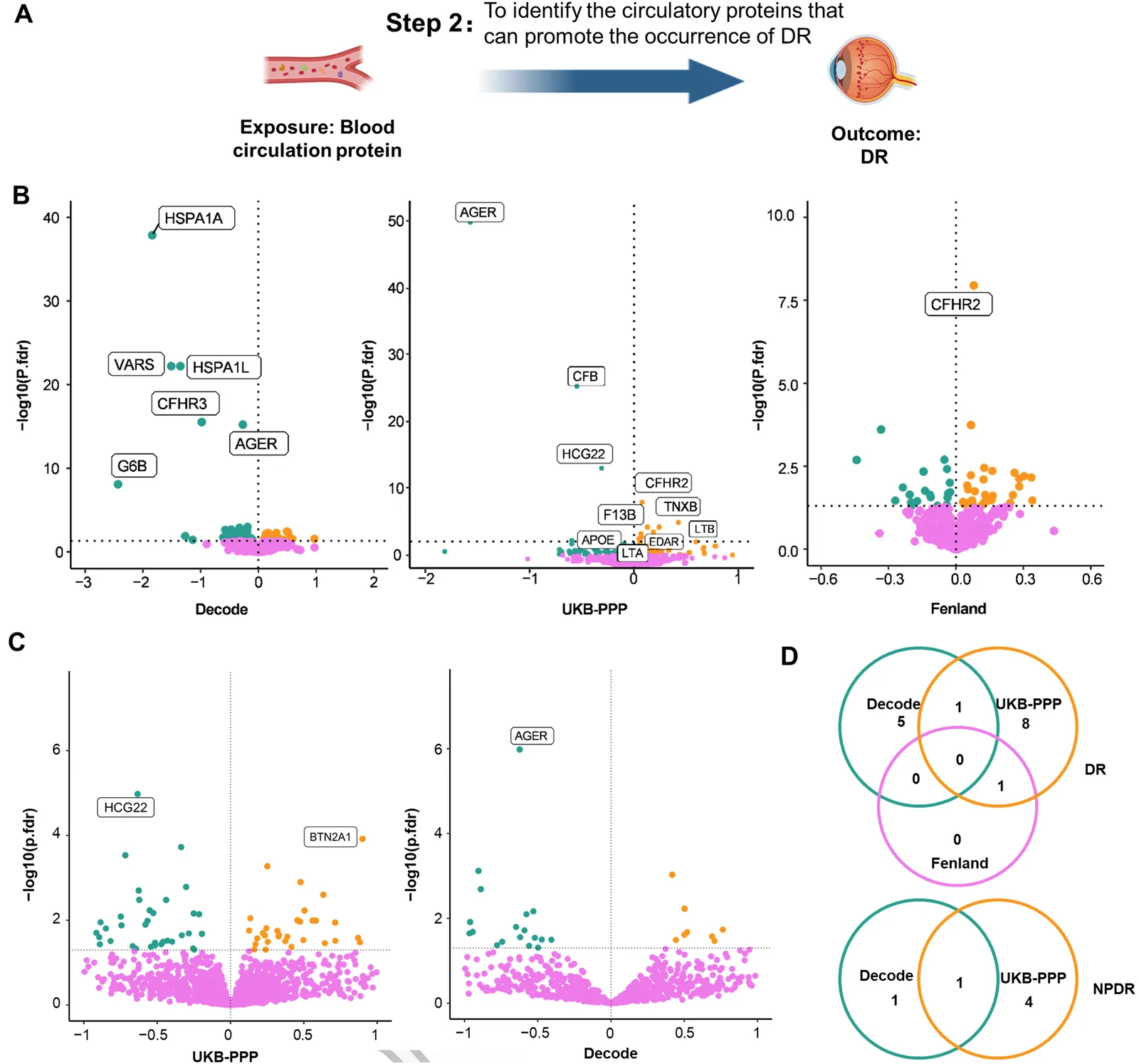
Proteome-wide MR analysis revealing proteins associated with DR. **(A)** Schematic diagram of exposure and outcome in proteome Mendelian randomization. **(B)** Nominally significant proteins associated with DR in the Decode, UKB-PPP, and Fenland datasets. **(C)** Nominally significant proteins associated with NPDR in the Decode, UKB-PPP, and Fenland datasets. (LTB and AGER were excluded from the display range due to excessively large beta values in UKB-PPP; HSPA1A was excluded from the display range due to excessively large beta values in Decode) **(D)** Venn diagram of FDR-corrected significant proteins associated with DR and NPDR across all three datasets. HSPA1A, heat shock 70 kDa protein 1A; HSPA1L, heat shock 70 kDa protein 1-like;VARS, Valine tRNA ligase; CFHR3, complement factor H-related protein 3; G6B, megakaryocyte and platelet inhibitory receptor G6b; AGER, advanced glycosylation end product-specific receptor; CFB, complement factor B; HCG22, HLA complex group 22; F13B, coagulation factor XIII B chain; TNXB, tenascin XB; CFHR2, complement factor H-related protein 2; APOE, apolipoprotein E; EDAR, ectodysplasin A receptor; LTA, lymphotoxin-alpha; LTB, lymphotoxin-beta; BTN2A1, butyrophilin subfamily 2 member A1.

We examined associations between genetic predisposition to DR and blood protein levels across three proteome-wide association study datasets (Decode, UKB-PPP, Fenland) (**Figure 3A**). In the Decode dataset, 64 out of 4,719 proteins showed associations with DR susceptibility, with 6 proteins remaining statistically significant after FDR correction (**Figure 3B**, **Supplementary Table 4**). The UKB-PPP and Fenland datasets revealed 148 and 52 DR-associated proteins, respectively, of which 10 and 1 remained significant post-FDR correction (**Figure 3B**, **Supplementary Table 5** for UKB-PPP and **Supplementary Table 6** for Fenland). Intersection analysis across datasets identified ANGPTL1 as consistently associated with DR in all three cohorts. Although no proteins reached significance in all datasets after FDR correction, AGER emerged as a protective factor for DR in both Decode and UKB-PPP, while CFHR2 acted as a risk factor in UKB-PPP and Fenland (**Figure 3B**).

For subgroup analyses, PDR was excluded from further investigation due to its unconfirmed association with NAFLD in Section 3.1. We identified 95 NPDR-associated proteins in UKB-PPP (4 significant after FDR correction), 59 in Decode (2 significant post-FDR), and 48 in Fenland (none surviving FDR correction) (**Figure 3C**, **Supplementary Tables 4–6**). Cross-dataset analysis confirmed AGER as a consistent protective factor for NPDR in both Decode and UKB-PPP after FDR adjustment (**Figure 3D**, **Supplementary Tables 4–6**).

### 3.3 Identification of plasma proteomics regulating the liver-eye axis in metabolic syndrome

**Figure 4:**
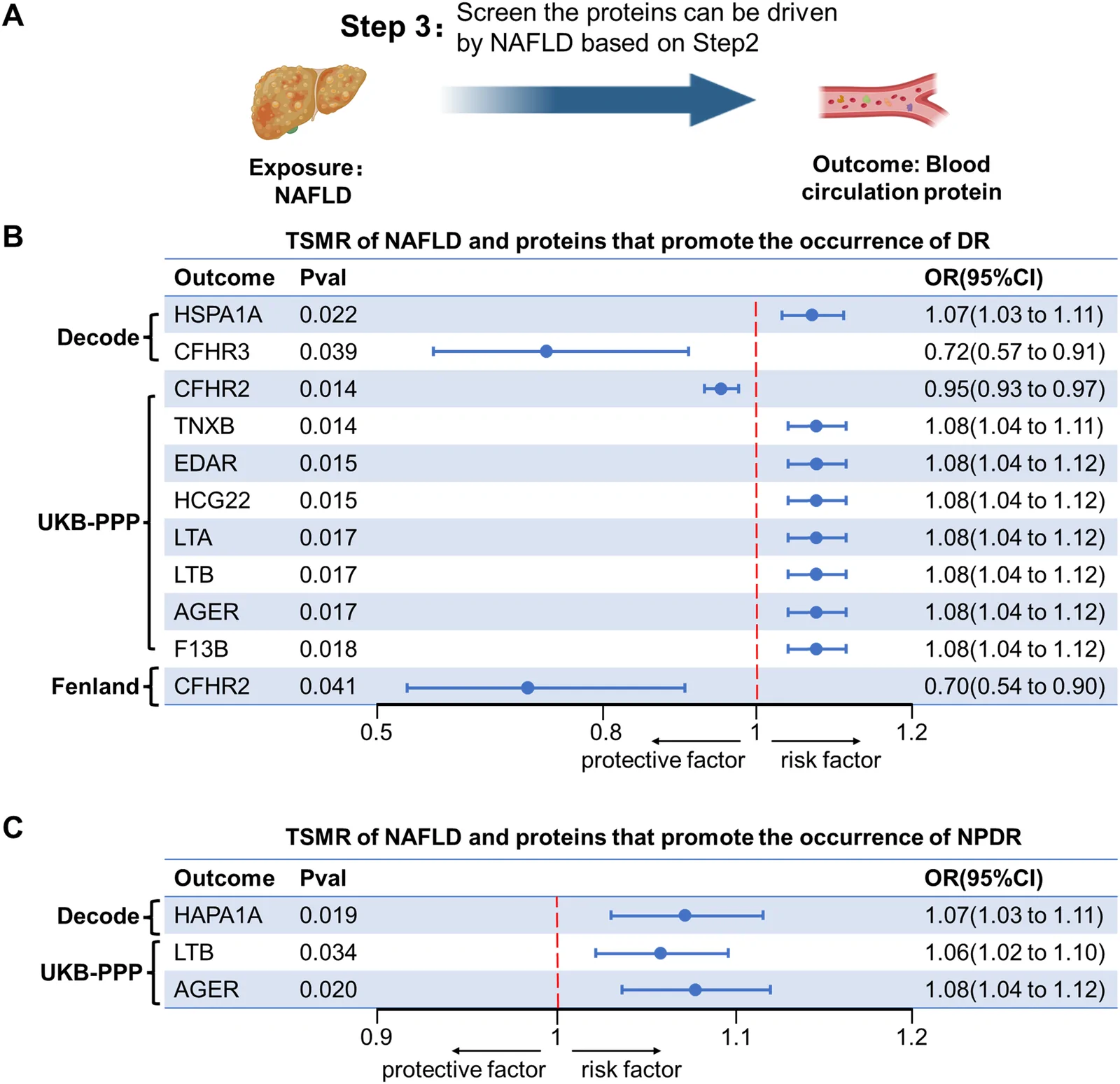
TSMR between NAFLD and DR-associated proteins. **(A)** Schematic diagram of exposure and outcome in two-sample Mendelian randomization. **(B)** Results of TSMR analysis between NAFLD and DR-associated proteins. **(C)** Results of TSMR analysis between NAFLD and NPDR-associated proteins. HSPA1A, heat shock 70 kDa protein 1A; CFHR3, complement factor H-related protein 3; CFHR2, complement factor H-related protein 2; AGER, advanced glycosylation end product-specific receptor; HCG22, HLA complex group 22; F13B, coagulation factor XIII B chain; TNXB, tenascin XB; EDAR, ectodysplasin A receptor; LTA, lymphotoxin-alpha; LTB, lymphotoxin-beta; CI, confidence interval; OR, odds ratio.

We subsequently conducted TSMR analyses to assess whether the FDR-corrected significant DR-associated proteins identified across three datasets (totaling 17 proteins) were influenced by NAFLD (**Figure 4A**). Results revealed significant associations between NAFLD and 10 proteins: HSPA1A(heat shock 70 kDa protein 1A), CFHR3(complement factor H-related protein 3), CFHR2(complement factor H-related protein 2), TNXB(tenascin XB), EDAR(ectodysplasin A receptor), HCG22(HLA complex group 22), LTA(lymphotoxin-alpha), LTB(lymphotoxin-beta), AGER(advanced glycosylation end product-specific receptor), and F13B(coagulation factor XIII B chain) (**Figure 4B**, **Supplementary Table 7**). Intersection analysis showed CFHR2 was significant in two datasets. Specifically, NAFLD promoted upregulation of HSPA1A, TNXB, EDAR, HCG22, LTA, LTB, AGER, and F13B (8 proteins), while downregulated CFHR3 and CFHR2.

Additionally, we evaluated NAFLD’s effects on NPDR-associated proteins (6 proteins analyzed). Significant associations were observed between NAFLD and three proteins: HSPA1A, LTB, and AGER (**Figure 4C**, **Supplementary Table 7**). The expression levels of these three plasma proteins were upregulated under the influence of NAFLD.

### 3.4 Colocalization analysis of blood proteins mediating liver-eye axis in metabolic syndrome

We performed colocalization analysis on blood proteins identified as being both NAFLD-driven and influencing DR/NPDR phenotypes to strengthen evidence for their mediating roles between NAFLD and DR. To qualify as mediators, proteins must either: upregulated by NAFLD and act as DR risk factors, or downregulated by NAFLD and act as DR protective factors. Among the 10 DR-associated proteins, 4 met these criteria and were analyzed. Only LTB showed strong colocalization evidence, while LTA, EDAR and TNXB demonstrated weak evidence (**Table 1**). For NPDR-associated proteins, only LTB fulfilled the inclusion criteria, exhibiting moderate colocalization evidence (**Table 1**).

**Table 1:** Co-localization analysis of blood proteins with mediating effects.

Source	Protein	PP.H0.abf	PP.H1.abf	PP.H2.abf	PP.H3.abf	PP.H4.abf
*DR-UKB*	LTA	0.000	0.000	0.000	1.000	0.000
*DR-UKB*	LTB	0.000	0.000	0.000	0.000	1.000
*DR-UKB*	EDAR	0.000	0.475	0.000	0.511	0.014
*DR-UKB*	TNXB	0.000	0.000	0.000	1.000	0.000
*NPDR-UKB*	LTB	0.000	0.479	0.000	0.000	0.521

### 3.5 Mediation effect analysis of blood proteins mediating liver-eye axis in metabolic syndrome

**Figure 5:**
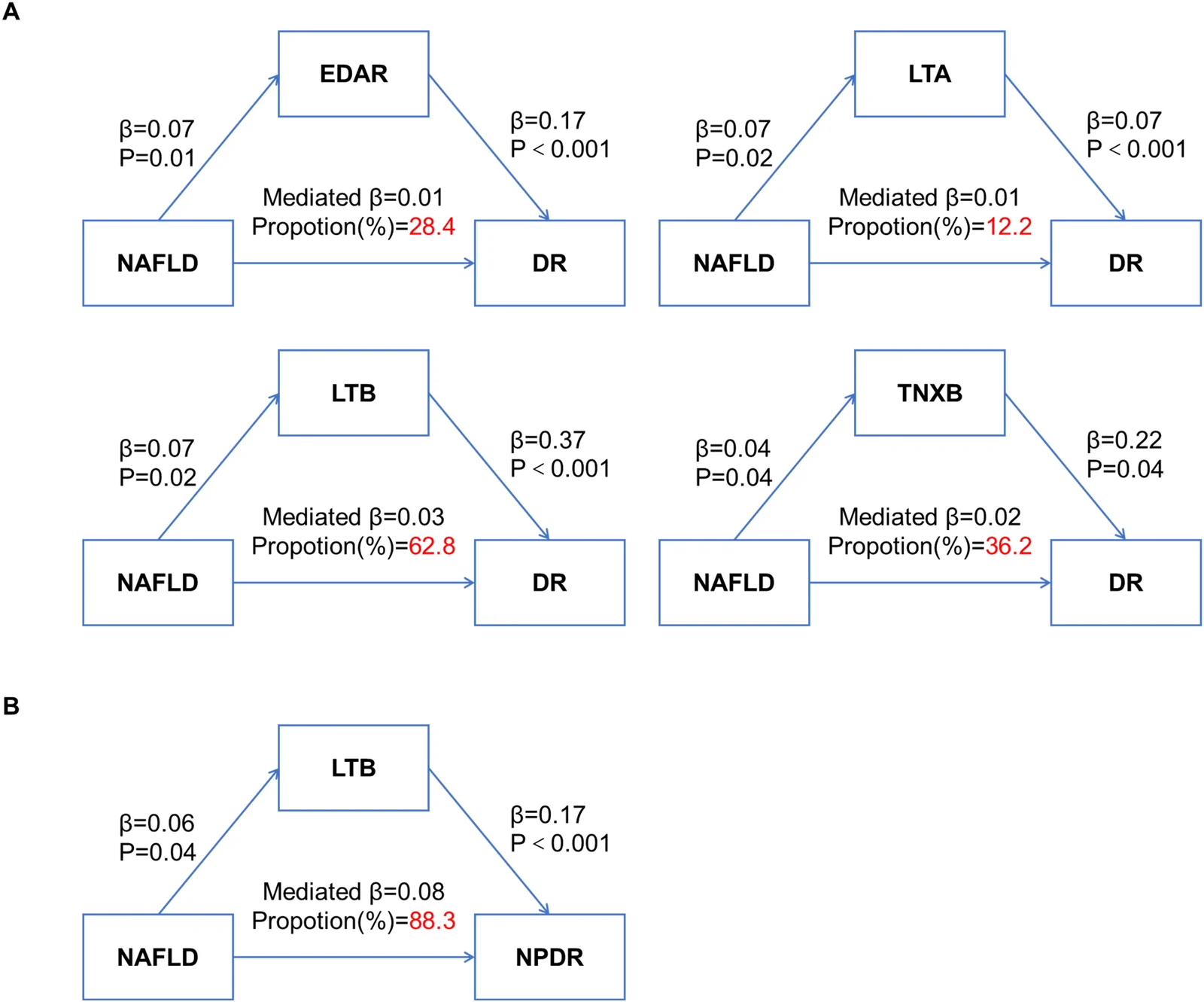
Mediation Mendelian randomization. **(A)** Mediation Effect Evaluation of Blood Proteins Mediating NAFLD-DR Associations. **(B)** Mediation Effect Evaluation of Blood Proteins Mediating NAFLD-NPDR Associations. TNXB, tenascin XB; EDAR, ectodysplasin A receptor; LTA, lymphotoxin-alpha; LTB, lymphotoxin-beta; NAFLD, non-alcoholic fatty liver disease; DR, diabetic retinopathy; PDR, proliferative diabetic retinopathy; NPDR, non-proliferative background diabetic retinopathy.

To determine the strength of mediation effects of blood proteins linking liver and eye, we conducted mediation Mendelian analysis using the coefficient product method on “NAFLD-Protein-DR” pathways meeting positive associations in “exposure-outcome, exposure-mediator, mediator-outcome” relationships. Among 10 candidate pathways, 6 were excluded due to directional inconsistency, with the remaining 4 pathways all originating from UKB-PPP. The results indicate that LTB mediates the strongest association between non-alcoholic fatty liver disease and diabetic retinopathy, followed by TNXB, EDAR, and LTA, respectively (**Figure 5A**, **Supplementary Table 9**). For NPDR, only LTB demonstrated consistent effect directions, mediating the NAFLD-driven effect on NPDR development (**Figure 5B**, **Supplementary Table 9**). Moreover, the presence of NAFLD promotes the upregulation of the expression of these circulating proteins in the blood, and the upregulation of the expression of these blood proteins will further promote the occurrence of DR.

### 3.6 Biological detection and verification of the identified liver-eye axis signaling pathway

**Figure 6:**
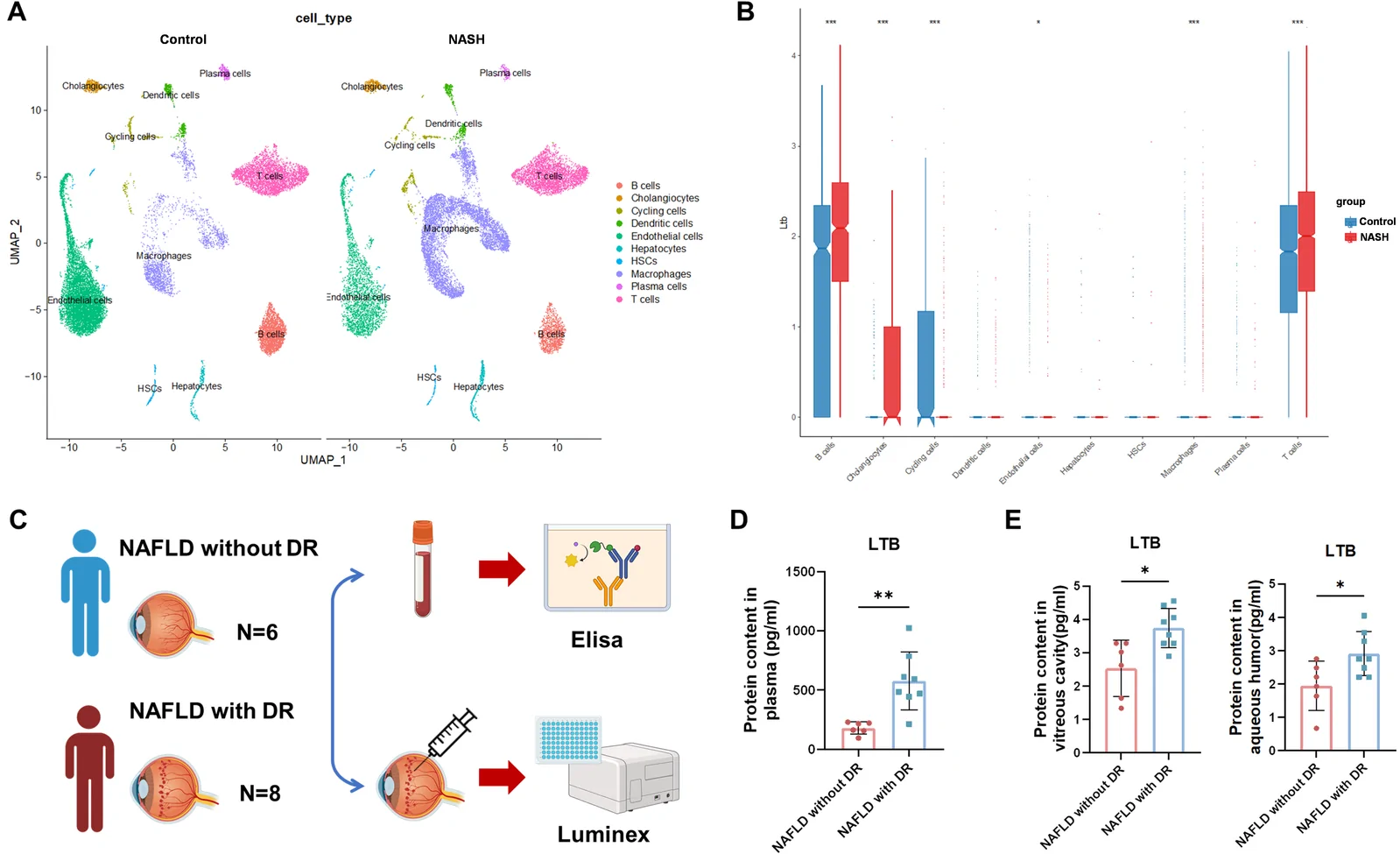
Identification of LTB protein expression in different biological samples along the liver-eye axis. **(A)** UMAP plots of cell clustering for single-cell transcriptome sequencing of livers from NASH mice and control mice. **(B)** The transcriptional expression levels of LTB in different cell types of the liver. **(C)** Schematic diagram of patient grouping and biological sample collection. **(D)** The ELISA test results of LTB protein expression levels in plasma. **(E)** The Luminex factor chip detection results of LTB protein expression levels in aqueous humor and vitreous humor. NAFLD, non-alcoholic fatty liver disease; DR, diabetic retinopathy; LTB, lymphotoxin-beta; *P < 0.05; **P < 0.01; ***P < 0.001.

To investigate whether LTB protein may serve as an intermediary in the liver-eye axis, we conducted further analyses using two complementary approaches. First, we reanalyzed the publicly available single-cell transcriptome dataset GSE129516 to examine LTB expression at the cellular level. Second, we measured LTB protein levels in clinical samples collected from patients. Initially, we analyzed the GSE129516 single-cell transcriptome sequencing dataset from the GEO database, which includes three NASH mouse samples and three normal mouse samples. Following dataset preprocessing, we classified the cells into ten distinct types (**Figure 6A**). Subsequent analysis of LTB expression across different cell types revealed upregulation in various liver cells, including B cells, cholangiocytes, and T cells (**Figure 6B**). Furthermore, LTB was predominantly highly expressed in T cells and B cells within the liver, with an overall increase in LTB expression levels in NASH mouse livers, providing supportive evidence for the potential cellular source of LTB. We collected plasma, vitreous humor, and aqueous humor samples from NAFLD patients with or without DR (**Figure 6C**). ELISA assays demonstrated higher LTB protein levels in the plasma of NAFLD patients with DR (**Figure 6D**). Additionally, for minute samples such as aqueous and vitreous humor, we employed Luminex liquid chip technology to measure LTB protein levels. The results indicated that NAFLD patients with DR exhibited higher LTB protein levels in both aqueous and vitreous humor compared to those without DR (**Figure 6E**). Collectively, these findings support the existence of a potential LTB-associated liver-eye axis, although the origin and trafficking of LTB require further investigation.

## 4 Discussion

Our research provides evidence supporting the existence of a liver-eye axis in metabolic syndrome, which may mediates the association between hepatic and ocular phenotypes. Specifically, the presence of NAFLD may facilitate the development of DR, particularly in the early non-proliferative stage, where hepatic abnormalities may already contribute to the emergence of ocular manifestations. Furthermore, we have explored a candidate mechanistic pathway underlying this liver-eye axis, mediated by circulating proteins. Hepatic phenotypes in metabolic syndrome promote the upregulation of specific plasma proteins, which could contribute to the development of ocular features associated with DR. Furthermore, we also identified LTB as a candidate protein that may link NAFLD to DR through the liver-eye axis pathway. Through single-cell sequencing, ELISA, and Luminex factor array assays, we have validated the expression levels of LTB at various stages within the proposed liver-eye axis signaling pathway, supporting the potential involvement of this pathway in linking hepatic and ocular pathologies.

In recent years, there has been a growing interest in the interaction between the liver and the eyes. The concept of the liver-eye axis describes the bidirectional connection between the liver and the eyes through metabolic pathways, molecular mediators and inflammatory responses, which is particularly significant in NAFLD and DR. Our previous study by using meta-analysis suggested that exist a positive correlation between NAFLD and DR induced by type 1 diabetes mellitus, and the presence of DR can reflect the progression of liver disease (6). The current research provides evidence supporting the existence of a potential liver-eye axis mediated by circulating proteins in NAFLD and DR. These findings offer a new perspective on disease progression beyond traditional single-organ-based approaches. Moreover, our findings suggest that NAFLD may be an independent risk factor for DR, raising the possibility that hepatic assessment in diabetic patients could aid in DR risk stratification. If validated in future prospective studies, this could facilitate earlier and more targeted intervention for patients at higher risk. In addition, past studies on the liver-eye axis have focused on the potential bridging role of molecular mediators (26). We also have identified four candidate plasma proteins that may link NAFLD and DR, providing potential biomarkers for risk stratification and early detection and intervention of DR and NAFLD.

A causal relationship between NAFLD and DR has been a controversial topic in previous studies. Most of the previous studies on this association were cross-sectional studies, which might have been influenced by various confounding factors, thus leading to inconsistent conclusions. G. Targher et al. found that in patients with type 1 and type 2 diabetes, the occurrence of NAFLD was positively correlated with the incidence of DR (27, 28). However, some studies have reported negative correlation between NAFLD and the risk of DR in the type 2 diabetes population (29–31). Additionally, a meta-analysis based on combined effect estimates did not find an overall association between NAFLD and the risk of DR in type 2 diabetes patients (32). Nevertheless, these studies were mostly observational and susceptible to residual confounding factors, including unmeasured risk factors, heterogeneity between studies, and selection/information bias. Therefore, in this Mendelian randomization study, we used randomly assigned genetic variations as instrumental variables to minimize confounding factors and ultimately provide evidence supporting causal relationship: NAFLD may promotes the development of DR. These findings add to the evidence base beyond previous observational studies. Our study is the first MR analysis to explore the relationship among NAFLD, DR and circulating proteins. One MR study investigated the association between NAFLD and three diabetic complications including DR but failed to observe a significant causal relationship between NAFLD and DR (33). Moreover, in addition to focusing on the relationship between diseases in previous studies, we integrated three major pQTL databases and conducted a systematic network MR analysis to provide a more systematic exploration of inter-organ communication and offer a proteomic perspective for the mechanism analysis of the complex cross-organ system regulation network of the liver-eye axis.

Our study has several methodological limitations that warrant consideration. First, the absence of detailed DR subclassification within the FinnGen dataset, specifically the lack of phenotypic stratification by diabetes type (T1DM and T2DM), precludes a comprehensive assessment of NAFLD’s differential causal effects on DR subtypes originating from distinct diabetic etiologies. Second, while exclusive use of European-ancestry data minimizes population stratification bias, it substantially constrains the generalizability of our findings to ethnically diverse populations. Third, the research primarily relies on large-scale population sequencing analyses, and the identified biological targets require further validation through comprehensive biological experiments. Fourth, the single-cell transcriptomic dataset GSE129516 is derived from a murine NASH model rather than uncomplicated NAFLD. Given that LTB is predominantly expressed by activated immune cells and that publicly available single-cell datasets of NAFLD with detailed immune cell subtyping are limited, we selected this dataset to explore the potential cellular source of LTB. While the NASH model was used to generate hypotheses about cellular origins, we performed protein-level validation directly in human NAFLD patients. Therefore, these single-cell findings should be interpreted as supportive and hypothesis-generating for the broader NAFLD population, rather than definitive proof. Lastly, the proposed LTB-mediated liver-eye axis requires more in-depth investigation, particularly regarding the mechanisms of LTB production in the NAFLD liver and its role in promoting DR progression in the ocular region. These aspects will be addressed in future studies.

## Data Availability

The original contributions presented in the study are included in the article/**Supplementary Material**. Further inquiries can be directed to the corresponding authors.

## References

[1] Huang DQ El-Serag HB Loomba R . Global epidemiology of NAFLD-related HCC: Trends, predictions, risk factors and prevention. Nat Rev Gastroenterol Hepatol. (2021) 18:223–38. doi: 10.1038/s41575-020-00381-6 33349658 PMC8016738

[2] Targher G Corey KE Byrne CD Roden M . The complex link between NAFLD and type 2 diabetes mellitus - Mechanisms and treatments. Nat Rev Gastroenterol Hepatol. (2021) 18:599–612. doi: 10.1038/s41575-021-00448-y 33972770

[3] Antonetti DA Silva PS Stitt AW . Current understanding of the molecular and cellular pathology of diabetic retinopathy. Nat Rev Endocrinol. (2021) 17:195–206. doi: 10.1038/s41574-020-00451-4 33469209 PMC9053333

[4] Moosaie F Davatgari RM Firouzabadi FD Esteghamati S Deravi N Meysamie A . Lipoprotein(a) and apolipoproteins as predictors for diabetic retinopathy and its severity in adults with type 2 diabetes: A case-cohort study. Can J Diabetes. (2020) 44:414–21. doi: 10.1016/j.jcjd.2020.01.007 32205075

[5] Yao X Pei X Fan S Yang X Yang Y Li Z . Relationship between renal and liver function with diabetic retinopathy in patients with type 2 diabetes mellitus: A study based on cross-sectional data. Sci Rep. (2022) 12:9363. doi: 10.1038/s41598-022-13164-7 35672376 PMC9174192

[6] Zhang G-H Yuan T-H Yue Z-S Wang L Dou G-R . The presence of diabetic retinopathy closely associated with the progression of non-alcoholic fatty liver disease: A meta-analysis of observational studies. Front Mol Biosci. (2022) 9:1019899. doi: 10.3389/fmolb.2022.1019899 36458094 PMC9706004

[7] Lu K Shi T-S Shen S-Y Shi Y Gao H-L Wu J . Defects in a liver-bone axis contribute to hepatic osteodystrophy disease progression. Cell Metab. (2022) 34 (3):441–57.e7. doi: 10.1016/j.cmet.2022.02.006 35235775

[8] Sun J-Y Du L-J Shi X-R Zhang Y-Y Liu Y Wang Y-L . An IL-6/STAT3/MR/FGF21 axis mediates heart-liver cross-talk after myocardial infarction. Sci Adv. (2023) 9:eade4110. doi: 10.1126/sciadv.ade4110 37018396 PMC10075967

[9] Yan L Yang F Wang Y Shi L Wang M Yang D . Stress increases hepatic release of lipocalin 2 which contributes to anxiety-like behavior in mice. Nat Commun. (2024) 15:3034. doi: 10.1038/s41467-024-47266-9 38589429 PMC11001612

[10] Wang F So K-F Xiao J Wang H . Organ-organ communication: The liver's perspective. Theranostics. (2021) 11:3317–30. doi: 10.7150/thno.55795 33537089 PMC7847667

[11] Kurki MI Karjalainen J Palta P Sipilä TP Kristiansson K Donner KM . FinnGen provides genetic insights from a well-phenotyped isolated population. Nature. (2023) 613:508–18. doi: 10.1038/s41586-022-05473-8 36653562 PMC9849126

[12] Kim DS Jackson AU Li YK Stringham HM Kuusisto J Kangas AJ . Novel association of TM6SF2 rs58542926 genotype with increased serum tyrosine levels and decreased APOB-100 particles in Finns. J Lipid Res. (2017) 58:1471–81. doi: 10.1194/jlr.P076034 28539357 PMC5496043

[13] Harder MN Ribel-Madsen R Justesen JM Sparsø T Andersson EA Grarup N . Type 2 diabetes risk alleles near BCAR1 and in ANK1 associate with decreased β-cell function whereas risk alleles near ANKRD55 and GRB14 associate with decreased insulin sensitivity in the Danish Inter99 cohort. J Clin Endocrinol Metab. (2013) 98:E801–6. doi: 10.1210/jc.2012-4169 23457408

[14] Frayling TM Timpson NJ Weedon MN Zeggini E Freathy RM Lindgren CM . A common variant in the FTO gene is associated with body mass index and predisposes to childhood and adult obesity. Science. (2007) 316:889–94. doi: 10.1126/science.1141634 17434869 PMC2646098

[15] Eldjarn GH Ferkingstad E Lund SH Helgason H Magnusson OT Gunnarsdottir K . Large-scale plasma proteomics comparisons through genetics and disease associations. Nature. (2023) 622:348–58. doi: 10.1038/s41586-023-06563-x 37794188 PMC10567571

[16] Argentieri MA Xiao S Bennett D Winchester L Nevado-Holgado AJ Ghose U . Proteomic aging clock predicts mortality and risk of common age-related diseases in diverse populations. Nat Med. (2024) 30:2450–60. doi: 10.1038/s41591-024-03164-7 39117878 PMC11405266

[17] Pietzner M Wheeler E Carrasco-Zanini J Cortes A Koprulu M Wörheide MA . Mapping the proteo-genomic convergence of human diseases. Science. (2021) 374:eabj1541. doi: 10.1126/science.abj1541 34648354 PMC9904207

[18] Hemani G Zheng J Elsworth B Wade KH Haberland V Baird D . The MR-Base platform supports systematic causal inference across the human phenome. Elife. (2018) 7:e34408. doi: 10.7554/eLife.34408 29846171 PMC5976434

[19] Burgess S Thompson SG . Interpreting findings from Mendelian randomization using the MR-Egger method. Eur J Epidemiol. (2017) 32:377–89. doi: 10.1007/s10654-017-0255-x 28527048 PMC5506233

[20] Bowden J Spiller W Del Greco MF Sheehan N Thompson J Minelli C . Improving the visualization, interpretation and analysis of two-sample summary data Mendelian randomization via the radial plot and radial regression. Int J Epidemiol. (2018) 47:2100. doi: 10.1093/ije/dyy265 30423109 PMC6280936

[21] Bowden J Davey Smith G Burgess S . Mendelian randomization with invalid instruments: Effect estimation and bias detection through Egger regression. Int J Epidemiol. (2015) 44:512–25. doi: 10.1093/ije/dyv080 26050253 PMC4469799

[22] Bowden J Davey Smith G Haycock PC Burgess S . Consistent estimation in Mendelian randomization with some invalid instruments using a weighted median estimator. Genet Epidemiol. (2016) 40:304–14. doi: 10.1002/gepi.21965 27061298 PMC4849733

[23] Hartwig FP Davey Smith G Bowden J . Robust inference in summary data Mendelian randomization via the zero modal pleiotropy assumption. Int J Epidemiol. (2017) 46:1985–98. doi: 10.1093/ije/dyx102 29040600 PMC5837715

[24] Glickman ME Rao SR Schultz MR . False discovery rate control is a recommended alternative to Bonferroni-type adjustments in health studies. J Clin Epidemiol. (2014) 67:850–7. doi: 10.1016/j.jclinepi.2014.03.012 24831050

[25] Geng J Ruan X Wu X Chen X Fu T Gill D . Network Mendelian randomisation analysis deciphers protein pathways linking type 2 diabetes and gastrointestinal disease. Diabetes Obes Metab. (2025) 27:866–75. doi: 10.1111/dom.16087 39592890 PMC7617254

[26] Yuan T-H Yue Z-S Zhang G-H Wang L Dou G-R . Beyond the liver: Liver-eye communication in clinical and experimental aspects. Front Mol Biosci. (2021) 8:823277. doi: 10.3389/fmolb.2021.823277 35004861 PMC8740136

[27] Targher G Bertolini L Rodella S Zoppini G Lippi G Day C . Non-alcoholic fatty liver disease is independently associated with an increased prevalence of chronic kidney disease and proliferative/laser-treated retinopathy in type 2 diabetic patients. Diabetologia. (2008) 51:444–50. doi: 10.1007/s00125-007-0897-4 18058083

[28] Targher G Bertolini L Chonchol M Rodella S Zoppini G Lippi G . Non-alcoholic fatty liver disease is independently associated with an increased prevalence of chronic kidney disease and retinopathy in type 1 diabetic patients. Diabetologia. (2010) 53:1341–8. doi: 10.1007/s00125-010-1720-1 20369224

[29] Kim B-Y Jung C-H Mok J-O Kang SK Kim C-H . Prevalences of diabetic retinopathy and nephropathy are lower in Korean type 2 diabetic patients with non-alcoholic fatty liver disease. J Diabetes Investig. (2014) 5:170–5. doi: 10.1111/jdi.12139 24843757 PMC4023580

[30] Yan L-H Mu B Guan Y Liu X Zhao N Pan D . Assessment of the relationship between non-alcoholic fatty liver disease and diabetic complications. J Diabetes Investig. (2016) 7:889–94. doi: 10.1111/jdi.12518 27181828 PMC5089952

[31] Afarideh M Aryan Z Ghajar A Ganji M Ghaemi F Saadat M . Association of non-alcoholic fatty liver disease with microvascular complications of type 2 diabetes. Prim Care Diabetes. (2019) 13:505–14. doi: 10.1016/j.pcd.2019.03.009 31054837

[32] Song D Li C Wang Z Zhao Y Shen B Zhao W . Association of non-alcoholic fatty liver disease with diabetic retinopathy in type 2 diabetic patients: A meta-analysis of observational studies. J Diabetes Investig. (2021) 12:1471–9. doi: 10.1111/jdi.13489 33372390 PMC8354494

[33] Liu N Wang G Liu C Liu J Huang S Zhou Y . Non-alcoholic fatty liver disease and complications in type 1 and type 2 diabetes: A Mendelian randomization study. Diabetes Obes Metab. (2023) 25:365–76. doi: 10.1111/dom.14877 36181433

